# Development of a Bispecific Antibody Targeting CD30 and CD137 on Hodgkin and Reed-Sternberg Cells

**DOI:** 10.3389/fonc.2019.00945

**Published:** 2019-09-24

**Authors:** Sakthi Rajendran, Yating Li, Evelyn Ngoh, Hiu Yi Wong, Man Si Cheng, Cheng-I Wang, Herbert Schwarz

**Affiliations:** ^1^Department of Physiology, National University of Singapore, Singapore, Singapore; ^2^NUS Immunology Programme, Life Sciences Institute, National University of Singapore, Singapore, Singapore; ^3^Singapore Immunology Network, Agency for Science, Technology and Research, Singapore, Singapore

**Keywords:** Hodgkin lymphoma, CD30, CD137, bispecific antibodies, immunotherapy

## Abstract

Hodgkin Lymphoma (HL) is a malignancy that frequently affects young adults. Although, there are effective treatments not every patient responds, necessitating the development of novel therapeutic approaches, especially for relapsed and refractory cases. The two TNF receptor family members CD30 and CD137 are expressed on Hodgkin and Reed Sternberg (HRS) cells, the malignant cells in HL. We found that this co-expression is specific for HRS cells. Based on this discovery we developed a bispecific antibody that binds preferentially to the CD30, CD137-double positive HRS cells. The CD30, CD137 bispecific antibody gets internalized into HRS cells opening up the possibility to use it as a carrier for a toxin. This antibody also induces antibody-dependent, cell-mediated cytotoxicity in CD30, CD137-double positive HRS cells. The enhances specificity of the CD30, CD137 bispecific antibody to HRS cells makes it a promising candidate for development as a novel HL treatment.

## Introduction

Hodgkin Lymphoma (HL) is a hematological malignancy that affects not only elderly but a significant number of young adults. For 2018, there are an estimated 8,260 new cases of HL and 1,070 deaths due to HL in the USA alone (1). Though, most HL patients respond well to conventional chemotherapy, relapses occur in around in 10 and 40% of early stage and late stage cases, respectively (2). High dose chemotherapy and autologous stem cell transplantation may help those patients but these treatments are associated with significant morbiditiy and mortality (3). With the emergence of checkpoint inhibitors, the objective response rates of HL has improved to above 60% (4). However, about 2% of patients fail to respond and about 13% will relapse after initial treatment (5).

Another treatment option is an anti-CD30 antibody conjugated to auristatin E (Brentuximab vedotin). CD30 is expressed on Hodgkin and Reed-Sternberg (HRS) cells, the malignant cells in HL. HRS cells constitute a small minority of about 1% in the HL tumor mass but they organize the huge inflammatory infiltrate and tumor microenvironment that is characteristic of HL (6, 7). Eliminating the HRS cells based on their CD30 expression clearly provides clinical benefit but Brentuximab vedotin is associated with toxicities (8), and more than 10% of patients relapse (9).

CD30 (TNFRSF8) and CD137 (TNFRSF9, 4-1BB) are members of the tumor necrosis factor receptor family. Like CD30, CD137 is also expressed on HRS cells, and 86% of HL cases are found to have CD137-expressing HRS cells (10, 11).

Ectopic expression of CD137 is induced by Epstein-Barr virus via the viral LMP-1 protein and helps HL to escape immune surveillance (12, 13). CD137 can be transferred by trogocytosis to CD137 ligand (CD137L)-expressing antigen presenting cells which leads to a complex formation of CD137 and CD137L and the internalization and degradation of CD137L. Since CD137-CD137L interaction is a main driver of type cellular immune response, the elimination of CD137L weakens an anti-HL T cell response (11). In addition, CD137 signals into HRS cells, inducing the secretion of IL-13, a potent growth factor for HL (14). IL-13 is a cytokine that deviates the immune response away from a protective type 1 toward a tumor-supporting type 2 response (15).

CD30 is not only expressed on HRS cells but also on activated T cells and that likely contributes to the toxicity of Brentuximab vedotin. Similarly, CD137 is expressed on activated T cells, NK cells, follicular dendritic cells, and vascular endothelial cells. But we wondered whether targeting CD30 and CD137 simultaneously with a bispecific antibody may enhance the specificity of an antibody based HL treatment. This enhanced specificity would result from the lack of avidity if only one arm of the antibody binds to CD30 which would be the case for CD30-single positive cells. However, for the Hodgkin and Reed Sternberg (HRS) cells that express CD30 and CD137, both antibody arms would bind, resulting in a much stronger binding of the antibody.

Here, we report on the generation of a bispecific antibody that targets CD30 and CD137. This bispecific antibody shows enhanced binding and antibody-dependent, cell-mediated cytotoxicity (ADCC) to CD30, CD137-double positive cells, which designates it as a promising candidate for a more efficacious and safer HL treatment.

## Materials and Methods

### Generation of Antibodies

Both anti-CD30 and anti-CD137 antibodies were isolated by *in vitro* biopanning from a human Fab phage display library that has a diversity of 30 billion clones (16). The experimental procedures for selection, phage preparation, Fab expression and purification closely followed the protocols described by De Haard et al. (17). Target proteins (R&D Systems, Minneapolis, MN, USA) were biotinylated using the EZ-Link NHS-PEG4-Biotin labeling kit (Pierce, Thermo Fisher Scientific, Waltham, USA). Biotinylated targets were first immobilized on M280 streptavidin-coated magnetic beads (Life Technologies Carlsbad, CA, USA). In the first round of biopanning, 10^13^ cfu phage were mixed with the bead-immobilized target in 1 ml casein-PBS blocking buffer; in the second and third rounds of biopanning, 10^11^ cfu phage were used in 0.5 ml blocking buffer. The phage library/target were incubated for 1 h at room temperature and the beads were washed with the PBS buffer containing 0.1% Tween-20. The concentrations of target proteins used in the biopanning step were 100, 20, and 5 nM, and the number of washes were 5, 10, and 25 times in rounds 1, 2, and 3, respectively. Bound phage were recovered by incubating the washed beads in 0.1 M triethylamine (pH 11) for 10 min followed by neutralization with 1 M Tris-HCl, pH 8. The recovered phages upon each round of biopanning were amplified. Following three rounds of biopanning, the selected Fab clones were expressed in *Escherichia coli* TG1 cells (Stratagene, Agilent Technologies, Santa Clara, California, USA) to screen for CD30 or CD137 binders by ELISA. Target binding unique clones were identified by DNA fingerprinting technique (17) and confirmed by DNA sequencing. To express full length IgG, the sequences of variable regions were cloned into human IgG1 backbone in the pTT5 vector. The anti-CD30/anti-CD137 bispecific antibodies were generated in CrossMab format (18), in which the variable region sequences of the heavy chain of anti-CD30 and anti-CD137 antibodies were cloned in the knob arm and hole arm, respectively. All full length antibodies were produced in HEK293-6E cells (19). Both the cells and the vector were obtained from National Research Council of Canada. Antibodies were purified from the culture supernatant using Protein G resin (Merck Millipore) following standard protocols.

### Cells

The HRS cell lines L-428, L-1236, KM-H2, and HDLM-2 were purchased from the German Collection of Microorganisms and Cell Cultures (DSMZ, Braunschweig, Germany) (20), and were cultured in RPMI 1640 (Sigma, St. Louis, MO, USA) supplemented with 10 or 20% fetal bovine serum (Biowest, Kansas City, MO, USA) at 37°C with 5% CO_2_. Stable cell lines expressing CD137 were generated by lentiviral transduction (11).

KM-H2 mutant cells were generated by deletion of CD30 or CD137 or both by transduction with lentiviruses expressing the sgRNA sequences 5′GTCGGTGACAGAACCCGTCG and 5′GCGCTGGAGAAACTATTTGG for CD30 and CD137, respectively.

Buffy coats from healthy donors were obtained from the National University Hospital, Singapore. Human PBMCs were isolated by density gradient centrifugation using Ficoll-Paque Plus (GE Healthcare, Chalfont St. Giles, UK). The protocol was approved by the National University of Singapore (NUS) IRB number B15-320E.

NK cells were isolated from PBMC by negative selection using EasySep™ Human NK Cell Enrichment Kit (Stemcell Technologies, Vancouver, Canada). 10^8^ PBMC were resuspended with Stem cell separation buffer (2% fetal bovine serum and 1 mM EDTA) in PBS. PBMC were incubated with NK enrichment antibody cocktail for 10 min, followed by incubation with Magnetic D beads in a polystyrene tube for 5 min. The tube was placed in EasySep magnet for 2.5 min and unlabeled cells were poured out from the tube. Enriched NK cells were resuspended in RPMI with 10% FBS and Pen-Strep.

### ADCC Assay

The ADCC assay was carried out using the Delfia EuTDA cytotoxicity kit (PerkinElmer, Waltham, MA, USA) according to manufacturer's instruction. In brief, target HRS cells (L-428-control or L-428-CD137 or L-1236-control or L-1236-CD137) were washed with RPMI with 10% FBS (R10) once and loaded with DELFIA BATDA reagent at 1 μl per 10^6^ cells in R10 supplemented with 2 mM probenecid and 50 μM β-mercaptoethanol (R10 PS PM media) for 20 min at 37°C. Labeled target cells were washed 4 times in R10 PS PM media, and were seeded into “V”-bottom 96-well plates at 10^4^ cells per well together with 5 μg/ml of bispecific or control antibodies for 40 min at 4°C. Effector cells (isolated NK cells) were added to the target cells at an E:T ratio of 20:1 and incubated for 3 h at 37°C. The Europium substrate solution was added to the supernatants for 15 min before the fluorescence was measured using a time-resolved fluorometer (VICTOR^3^ multi-label reader, PerkinElmer).

### Internalization Assays

KM-H2 cells were treated with 10 μg/ml of control HA4 or bispecific antibodies on ice. Cells were washed twice with ice-cold PBS and treated with 5 μg/ml of anti-human Fc-Biotin for 10 min on ice to crosslink the antibodies on cell surface. Cells were washed and incubated at 37°C with media for several time points. Cells were stained with streptavidin-APC and analyzed by Fortessa Analyzer (BD Biosciences, New Jersey, USA).

Internalization was further analyzed by treating KM-H2 cells with 50 μM of monodansylcadaverine (MDC, Sigma-Aldrich). The presence of the bispecific antibodies was analyzed 24 h later by flow cytometry.

### Multiplex Immunoflourescence Staining

Five micrometer tissue microarray slides containing 48 cases of HL (HL481) were purchased from US Biomax, Inc. (Derwood, MD, USA). Opal multiplex immunofluorescent system (opal staining) was adopted for multi-color staining (Perkin Elmer, Waltham, USA). Sections were incubated with the primary antibodies mouse anti-human CD137 (clone BBK-2, Thermo Fisher Scientific), rabbit anti-human CD3 (Dako), and rabbit anti-human CD30 (clone EPR4102, Abcam), followed by the secondary antibodies anti-mouse and anti-rabbit horseradish peroxidase (HRP) polymers (GBI Labs, Bothell, USA), and then developed with the 4-color Opal IHC kit (Perkin Elmer). Nuclei were stained with DAPI. Unstained sections of HL served as controls to collect autofluorescence signature for image unmixing. Images were acquired with the Vectra imaging system (Perkin Elmer) and analyzed with the inForm® advanced image analysis software (Perkin Elmer) at the Genome Institute of Singapore, A^*^STAR.

### Flow Cytometry

Cells were stained with PE-conjugated anti-CD137 antibody (clone 4B4-1, BD Biosciences) or FITC-conjugated anti-CD30 (clone BY88, Biolegend, San Diego, CA, USA) or mouse-IgG1k-PE (eBioscience, San Diego, USA) at 4°C in the dark for 15 min. Flow cytometry was performed with Fortessa flow cytometry (BD Biosciences) and analyzed with Flow Jo software (FlowJo, LLC, Ashland, USA). The rate of apoptosis was determined by Annexin V and 7-AAD staining (Biolegend).

### Statistics

Statistical significance was determined by a two-tailed unpaired Student's *t*-test.

## Results

### Co-expression of CD30 and CD137 on HRS Cells

**Figure 1:**
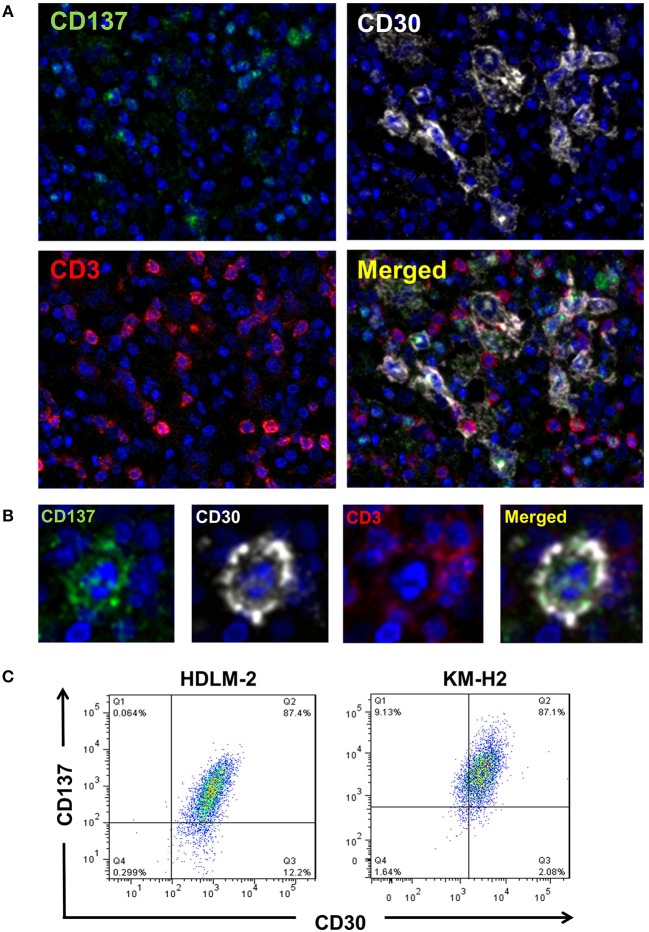
HRS cells express CD30 and CD137 in HL tissues. **(A)** HL tissue sections were stained by multiplex immunofluorescence for CD137 (green), CD30 (white), CD3 (red), and chromatin (blue). Magnification 100x. **(B)** One HRS cell enlarged. Same staining conditions as in **(B)**. **(C)** KM-H2 and HDLM-2 cells were stained for CD30 and CD137. Gatings are based on the isotype stained control for each cell line.

CD137 is expressed by HRS cells of 86% of classical HL tumor samples (10, 11). To test if HRS cells co-express CD30 and CD137, we performed multiplex immunoflourescence staining (Figures 1A,B). There is a clear co-localization of CD30 (white) and CD137 (green) on giant cells, confirming co-expression of the two molecules on HRS cells. The reason why the CD30 staining looks different from the CD137 stainings is that CD30 is mainly on the cell surface while most of CD137 is cytoplasmic. This is due to the fact that CD137 yields a complex with CD137 ligand which then gets internalized (11). T cells (red) present in the tumor stroma do not stain positive for CD137, an activation marker, indicating that the cells are not activated (21).

In addition, for the HRS-derived cell lines KM-H2 and HDLM-2 cells around 84 and 87%, respectively, of cells were CD30^+^CD137^+^ (Figure 1C). These data clearly demonstrate a co-expression of CD30 and CD137 on HRS cells.

### PBMC Do Not Co-express CD30 and CD137

Both, CD30 and CD137, can be expressed on activated lymphocytes. In order to evaluate whether a simultaneous targeting of CD30 and CD137 would also affect activated lymphocytes, we determined whether CD30 and CD137 are co-expressed on these cells.

**Figure 2:**
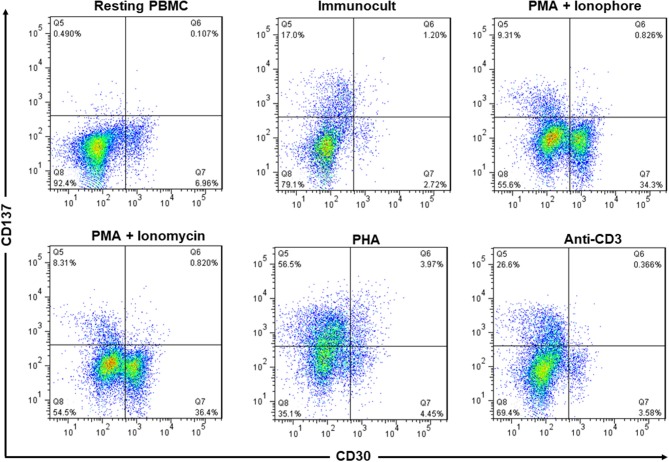
Expression of CD137 and CD30 are mutually exclusive in activated PBMC. PBMC from healthy donors were activated with immunocult, PMA (50 ng/ml) + ionophore (1 μM), PMA (50 ng/ml) + ionomycin (1 μg/ml), PHA (10 μg/ml), or anti-CD3 (5 ng/ml, clone OKT3) for 48 h to activate the lymphocytes. Cells were stained with 5 μg/ml of anti-CD137-PE and anti-CD30-FITC and analyzed by flow cytometry. Numbers in quadrants indicate percentages of cells. Data are representative of 2 independent experiments.

A small minority of resting PBMC stained positive for CD30 while no CD137 could be detected in unstimulated PBMC. Activation of PBMC by immunocult, PMA + ionophore, PMA + ionomycin, PHA, or anti-CD3 induced expression of CD30 or CD137 to various degrees but the cells expressed either CD30 or CD137 but not both together. Only in PHA-activated PBMC there was a small percentage of CD30^+^CD137^+^ cells (Figure 2). This finding confirms an earlier report that noticed a mutual exclusion of CD30 and CD137 expression on T cells (22), implying that co-expression of CD30 and CD137 is rather specific for HRS cells, and that targeting the two molecules would not affect activated lymphocytes.

### Generation and Testing of Anti-CD30 and Anti-CD137 Fabs

We generated 4 antigen binding Fab for anti-CD30 and anti-CD137 through phage display. Binding efficiency of the anti-CD30 and anti-CD137 Fabs were tested by dose response ELISA. The HL cell line L-428 homogenously expresses CD30 on its surface (Supplementary Figure 1). Hence, L-428 cells were treated with 10 μg/ml of anti-CD30 Fabs (clones 30A, 30B, and 30C) washed and probed with mouse anti-myc and anti-mouse-AF488. All Fabs efficiently bound to CD30 on the surface of L-428 cells. Fabs 30A and 30C showed a strong binding to CD30 with geometric mean fluorescence (GMF) of around 2,000 or more, while clone 30B had a moderate binding with GMF of around 1,000 (Supplementary Figure 2). We selected 30A and 30B for the anti-CD30 arm of the bispecific antibody as they had the least binding affinities thereby reducing the likelihood of the bispecific antibody binding to CD30-single positive cells.

The anti-CD137 Fabs 137A, 137B, 137C, and 137D were tested for their efficiency to bind to CD137-expressing HRS cell lines. CD137-transfected L-428 cells (L-428-CD137) were treated with 10 μg/ml of Fabs, followed by anti-Fab-AF647. All Fabs bound efficiently to CD137^+^ cells (Supplementary Figure 3). No binding was observed to CD137^−^ L-428-control cells (not shown). 137A and 137C showed weaker binding affinity than the other Fabs, making them suitable for the generation of the bispecific antibody.

### Testing the Functional Activity of Anti-CD137 Antibodies

**Figure 3:**
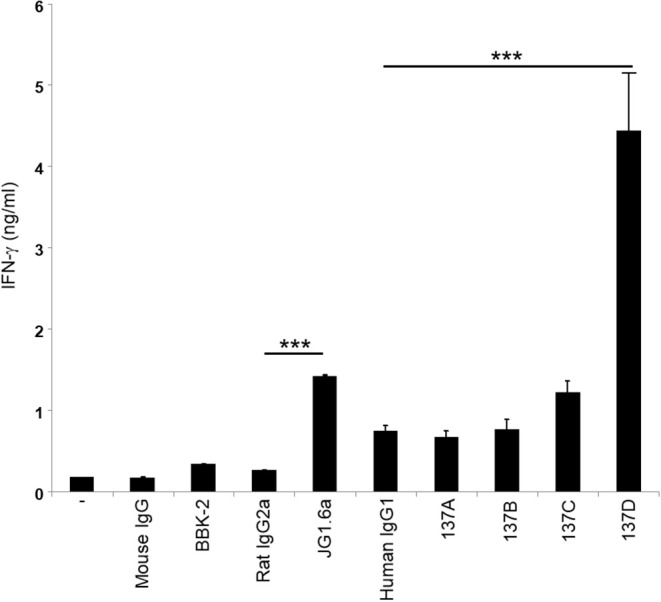
Characterization of the activity of anti-CD137 antibodies. Isolated T cells were cultured on plates coated with 5 μg/ml of anti-CD3 (clone OKT3) and 10 μg/ml of anti-CD137 antibodies for 48 h. IFN-γ levels were measured by ELISA. Means ± SD of triplicate measurements are depicted. ***p < 0.005. Data are representative of 3 independent experiments.

As CD137 signaling in HRS cells induces IL-13 release and thereby favors immune evasion, we aimed to select an antagonistic anti-CD137 arm for the generation of the bispecific antibodies. Hence, the Fabs were converted to full anti-CD137 antibodies and tested in a functional assay for co-stimulating T cells. T cells were seeded on wells coated with 5 μg/ml of anti-CD3 (clone OKT3) and 10 μg/ml of anti-CD137 antibodies. Anti-CD137 clone 137D increased secretion of IFN-γ suggesting it having an agonistic activity. The commercially available agonistic anti-CD137 antibody (clone JG1.6a) and antagonistic anti-CD137 antibody (clone BBK-2) have been used as controls. Treatment with 137A and 137B did not increase IFN-γ levels (Figure 3). The anti-CD137 Fab (137A) was selected for anti-CD137 arm of bispecific antibody, because it lacks agonistic activity and because of its moderate binding affinity to CD137-single positive cells.

### Binding Specificity of CD30, CD137-Bispecific Antibodies to HL Cell Lines

A bispecific antibody in CrossMab format (18) was generated with 137A as the anti-CD137 arm and 30B as the anti-CD30 arm, and its binding specificity was tested on HL cell lines, being single positive or double positive or double negative for CD30 and CD137 (Supplementary Figure 4). Although, this antibody preferentially bound to double-positive (CD30^+^CD137^+^) L-428-CD137 cells, it still retained binding to single positive cells (not shown). Therefore, this bispecific antibody was re-engineered by site directed mutagenesis to reduce the affinity of each arm. To this end, each tyrosine or tryptophan residue in the heavy and light chain CDR3 loops of these two antibodies was individually replaced with an alanine to create 6 single mutation bispecific antibody variants.

**Figure 4:**
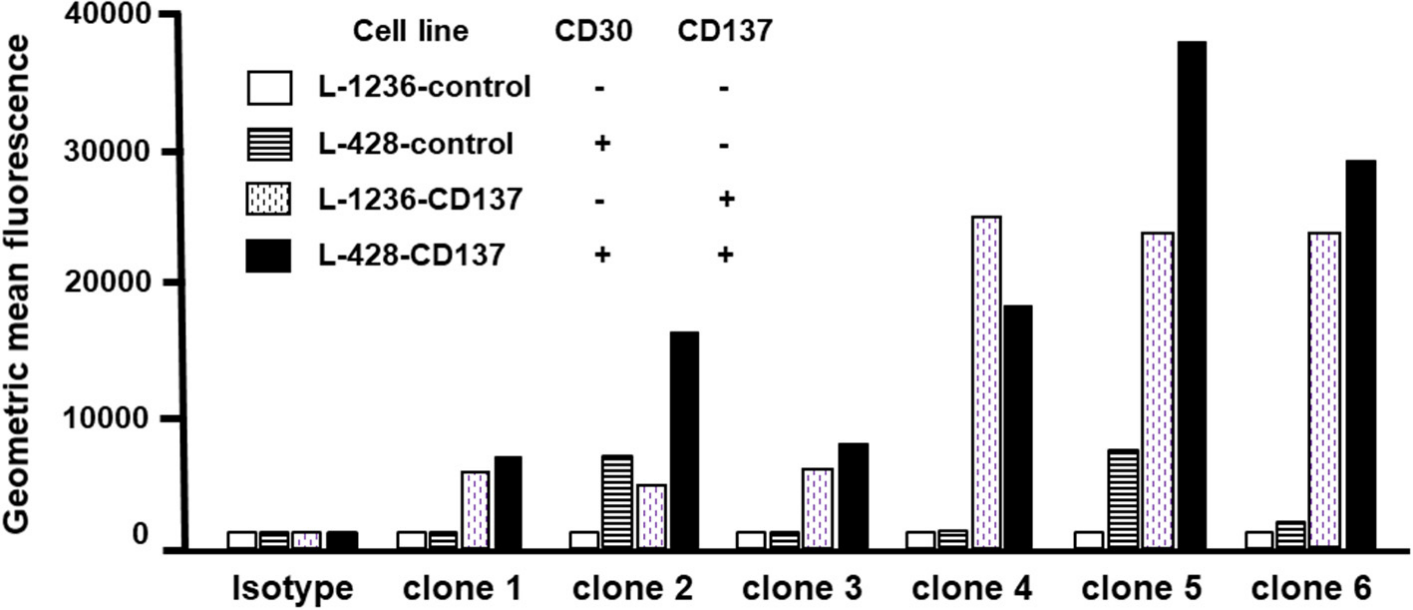
Binding of bispecific antibodies to HL cell lines. HL cells were treated with 5 μg/ml of bispecific antibodies (137A-30A, 137A-30B) or human IgG (Isotype) for 30 min at 4°C. Secondary staining was performed by anti-human Fc-PE and cells were analyzed by flow cytometry. Geometric Mean Fluorescence values are displayed in the bar chart. Data are representative of 2 independent experiments.

The binding specificity of 6 re-engineered clones was tested by flow cytometry. None of the clones bound double negative cells (white bars), and all bound to varying degrees single CD30- or CD137-positive cells (patterned bars). Clone 2 and 5 displayed highest binding to double-positive cells (black bars) (Figure 4). Affinities were measured by surface plasmon resonance. Clone 2 has a 9.3 nM and 16.3 nM affinity to CD30 and CD137, respectively; clone 5 has a 10.0 nM and 6.2 nM affinity to CD30 and CD137, respectively. These affinities are a few fold lower that that original 137A for CD137 (1.2 nM) and 30B for CD30 (2.7 nM). These two affinity-reduced bispecific antibodies were selected for functional analysis.

### Internalization Assay of Bispecific Antibodies

**Figure 5:**
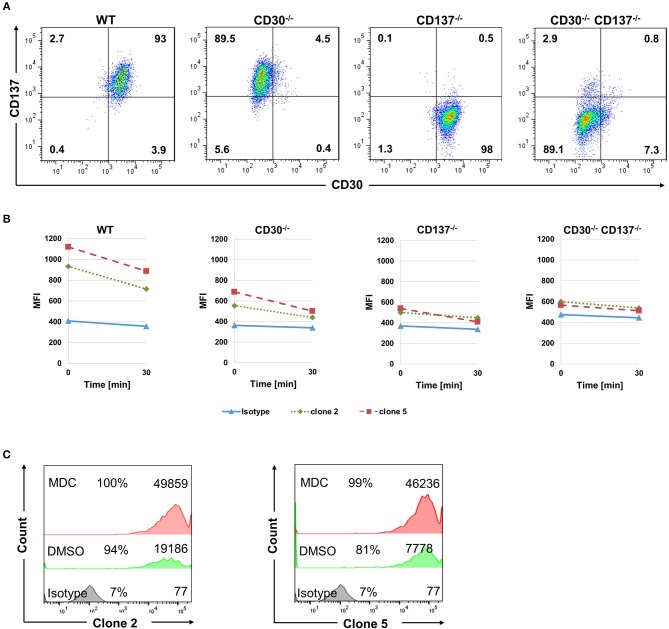
Bispecific antibodies are internalized into HL cells. **(A)** Expression of CD30 and CD137 on KM-H2 clones in which CD30 or CD137 or both were deleted. **(B)** WT (CD30^+^, CD137^+^), CD30-negative (CD30^−^, CD137^+^), CD137-negative (CD30^+^, CD137^−^), and double-negative (CD30^−^, CD137^−^) KM-H2 cells were treated with 10 μg/ml of biotinylated isotype control antibody or clones 2 and 5. Surface staining was detected by Streptavidin-APC after cross-linking the antibodies with anti-human Fc. Geometric mean fluorescence values of surface staining were represented. Data are representative of 3 independent experiments. **(C)** Same experimental set up as in **(B)** but KM-H2 were treated with 50 μM of monodansylcadaverine (MDC) or the solvent DMSO. Levels of surface-bound clones 2 and 5 were determined by flow cytometry after 24 h. The numbers in panels indicate the percentages of positive cells and the mean fluorescence intensity (MFI).

The FDA approved anti-CD30-drug conjugate exerts its therapeutic activity via being internalized and delivering the toxin auristatin E (23). We therefore tested if the bispecific antibodies can be internalized into HL cells, and whether that is dependent on CD30 and CD137. Binding of mutant 2 and 5 to the KM-H2 cells was almost abolished in the absence of either CD30 or CD137 as seen by the reduced MFI (Figure 5). *In vivo*, Fc receptors will crosslink the cell-bound antibodies and accelerate internalization of antibody. To simulate this *in vitro*, anti-Fc-Biotin was used to crosslink the antibodies. Internalization was measured by the reduction in the surface staining as a function of time. Both antibodies were internalized in WT cells. There was some residual internalization when only CD137 was present, i.e., in CD30^−/−^ cells, but internalization was absent in CD30, CD137 double negative cells (Figures 5A,B), demonstrating that these antibodies may be suitable as toxin carriers and specific to CD30, CD137 double positive HRS cells.

To further demonstrate internalization, we made use of Monodansylcadaverine (MDC), an inhibitor of internalization. Treatment of KM-H2 cells with 50 μM MDC increased the level of both bispecific antibodies on the surface of KM-H2 cells, demonstrating that internalization indeed takes place (Figure 5C). Internalization is likely mediated by the Fcγ receptor CD32a, since KM-H2 cells express CD32a while CD16 and CD64 could not be detected (Supplementary Figure 4).

### Killing Efficiency of Bispecific Antibodies

**Figure 6:**
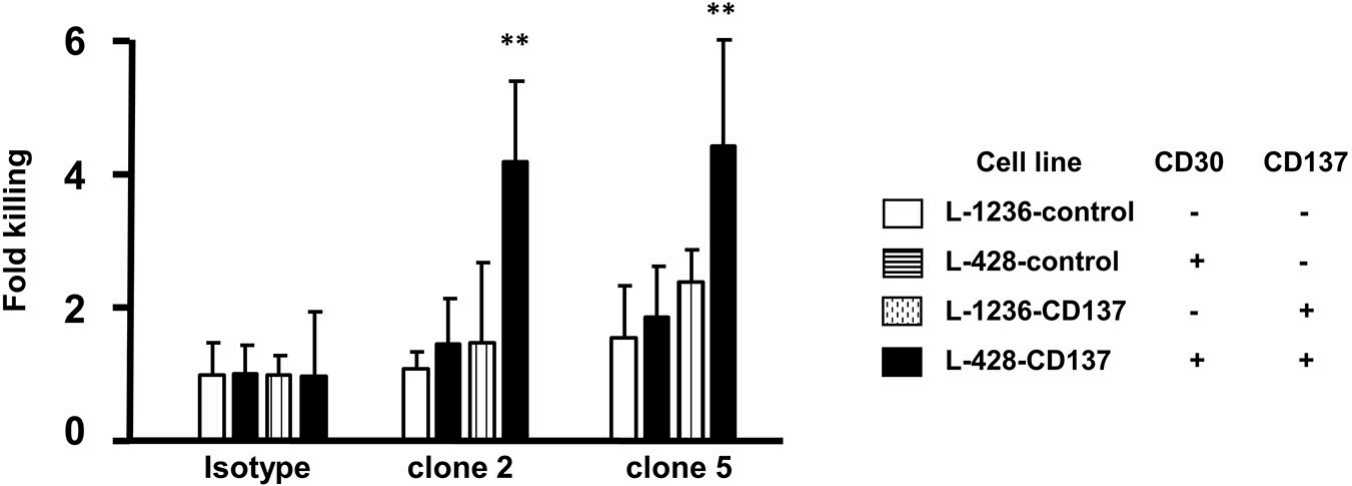
ADCC activity of bispecific antibodies. HL cells were labeled with 1 μl/ml of BATDA reagent and treated with 5 μg/ml of bispecific antibodies (137A-30A, 137A-30B) or human IgG (Isotype) for 40 min at 4°C. Effector NK cells were added at E:T of 20:1 and incubated for more than 3 h. Means ± SD of fold killing are represented (**p < 0.01). Data are representative of 3 independent experiments.

An alternative to killing HRS cells by an antibody drug conjugate is via immune effector functions such as ADCC. Therefore, clone 2 and 5 were tested for their efficiency to kill HL cells by ADCC using primary NK cells as effector cells. Spontaneous lysis of all 4 HL cell lines was normalized to 1. Treatment with clone 2 or 5 led to a 4-fold increase in the killing of double positive (CD30^+^CD137^+^) cells (black vs. white bars). Killing of single positive cells by clone 2 and 5 was close to spontaneous lysis levels (patterned bars compared to white bars) (Figure 6). This demonstrates the selectivity of the bispecific antibody to double positive (CD30^+^CD137^+^) cells.

### Direct Induction of Apoptosis

**Figure 7:**
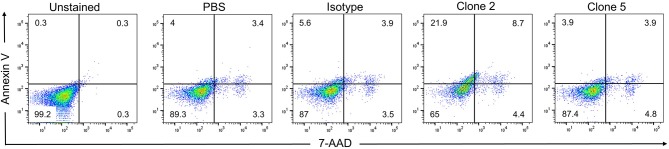
Clone 2 induces apoptosis. Antibodies were coated onto tissue culture plates at 5 μg/ml, washed and KM-H2 cells were added. After 24 h apoptosis was quantified by Annexin V and 7-AAD staining. Numbers in quadrants indicate percentages of cells. Data are representative of 2 independent experiments.

While drug-conjugated antibodies or antibodies that exert ADCC activity are useful anti-cancer agents, they require the use of toxins or the presence of NK cells, respectively. The ideal situation for a therapeutic antibody is to induce cell death directly upon binding to its target. While clone 5 showed no activity, clone 2 induced cell death in KM-H2 cells, increasing the rates of early and late apoptosis from 5.6 to 21.9% and from 3.9 to 8.7%, respectively, compared to the isotype control antibody (Figure 7). While clone 2 induced cell death in KM-H2 cells, we could not detect any change in cytokine levels (not shown).

## Discussion

These data demonstrate co-expression of CD30 and CD137 on HRS cells. Both, CD30 and CD137 are induced by EBV, and EBV may be the reason for co-expression of the two proteins in HRS cells. Both proteins are also expressed by certain immune cells upon activation but simultaneous expression does not occur in healthy cells. The mechanism for this mutual exclusion is not known. However, we think it is not a mutual inhibition as we do not see a change in CD30 levels if HL cells express CD30, and vice versa (Supplementary Figure 1 and unpublished data). This indicates that CD30 and CD137 expression mark different and likely non-overlapping activation states of T cells.

CD137 provides a growth advantage by reducing immune surveillance and by enhancing secretion of IL-13, a potent HRS cell growth factor. Since CD30 is also induced by EBV this indicates that CD30 may provide a growth and/or selection advantage as well. However, this has not yet been identified (24). As both proteins are co-stimulatory molecules it is plausible to assume that CD30 in an yet unidentified way supports HL growth.

This study shows that it is possible to enhance the specificity of an antibody to HRS cells by simultaneously targeting CD30 and CD137. The bispecific antibody was able to specifically induce ADCC in CD30^+^CD137^+^ cells, while causing minimal or no toxicity in single positive cells and double negative cells. Since this antibody is being internalized, it can be used as a drug or toxin carrier.

Since, we selected an anti-CD137 antibody that is not agonistic, the bispecific antibody will not induce CD137 signaling in HRS cells which would be expected to (1) enhance HRS cell proliferation, and (2) induce IL-13 secretion which may deviate a protective immune response toward a tumor-supporting Th2 response (15). Indeed, we could not detect any change in cytokine secretion in the HRS cell lines by the bispecific antibody. Although, it may change signaling, likely through the anti-CD30 arm since it enhanced apoptosis induction in KM-H2 cells. Further, binding of the bispecific antibody to CD137 on HRS cells would also prevent the trogocytic transfer of CD137 from HRS cells to adjacent APC which would lead to a downregulation of CD137L and a further inhibition of a protective type 1 response (11). Trogocytic transfer has recently also been shown for CD30 (25), and it is possible that this bispecific antibody also prevents CD30 trogocytosis.

This bispecific antibody can also be converted to a bispecific chimeric antigen receptor (CAR) in which one CAR delivers the T cell receptor signal while the other CAR delivers the co-stimulatory signal.

The increased specificity of this CD30, CD137 bispecific antibody, and the option to use it various therapeutic approaches, i.e., as a drug carrier or as an ADCC-inducing antibody or its employment in a CAR warrants its further development.

## Author Contributions

C-IW and HS designed the study. EN screened and expressed the antibodies. SR, YL, HW, and MC acquired the experimental data and performed the statistical analysis. SR and HS drafted the manuscript. All authors read and approved the final manuscript.

### Conflict of Interest

The authors declare that the research was conducted in the absence of any commercial or financial relationships that could be construed as a potential conflict of interest.
