# Supplementary material for: Development of a Bispecific Antibody Targeting CD30 and CD137 on Hodgkin and Reed-Sternberg Cells
Source: Front Oncol. 2019 Sep 24;9:945. doi: 10.3389/fonc.2019.00945 (PMC6768943; doi:10.3389/fonc.2019.00945)
Supplement: Supplementary file 1 [file Data_Sheet_1.PDF]

Supplementary Figure 1

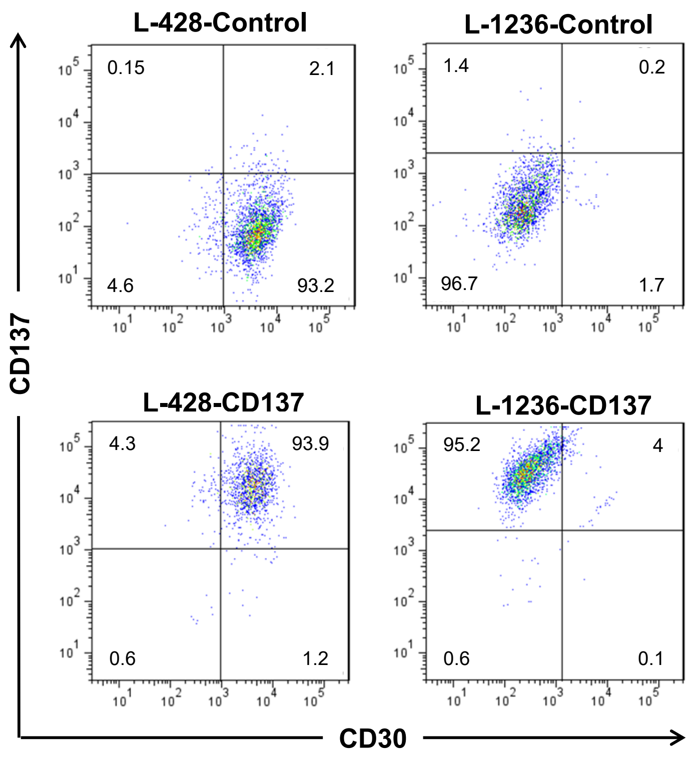

Cell line	CD30	CD137
L-1236-control	-	-
L-428-control	+	-
L-1236-CD137	-	+
L-428-CD137	+	+

Table 1. Expression profile of CD30 and CD137 in HL cell lines.
L-428-control or L-428-CD137 or L-1236-control or L-1236-CD137 cells were stained for CD30 and CD137 and analysed by flow cytometry. Numbers in quadrants indicate percentages of cells.

A

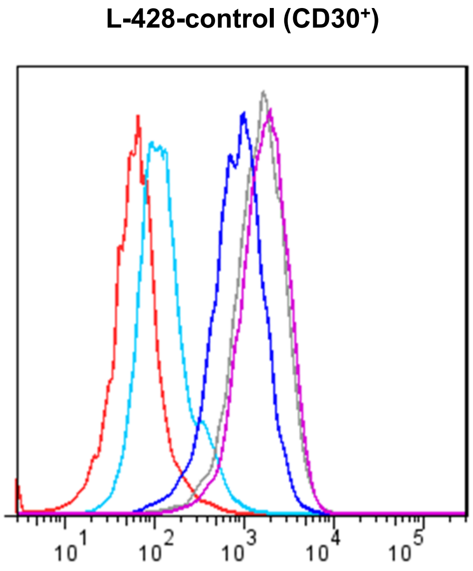

B

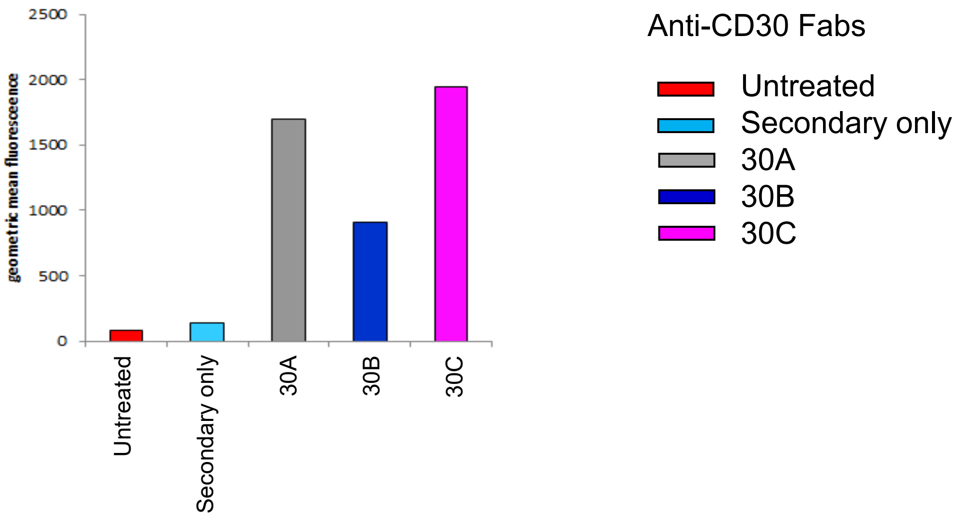

Binding of anti-CD30 Fabs to CD30+ HL cell line (L-428-control).
5 x 10^5 L-428-control cells were treated with 10 µg/ml of anti-CD30 Fabs and detected using anti-myc and anti-mouse-AF488. (A) Histogram of anti-CD30 Fab binding to L-428-control cells. (B) Geometric mean fluorescence of anti-C30 Fab binding. Data are representative of 2 independent experiments.

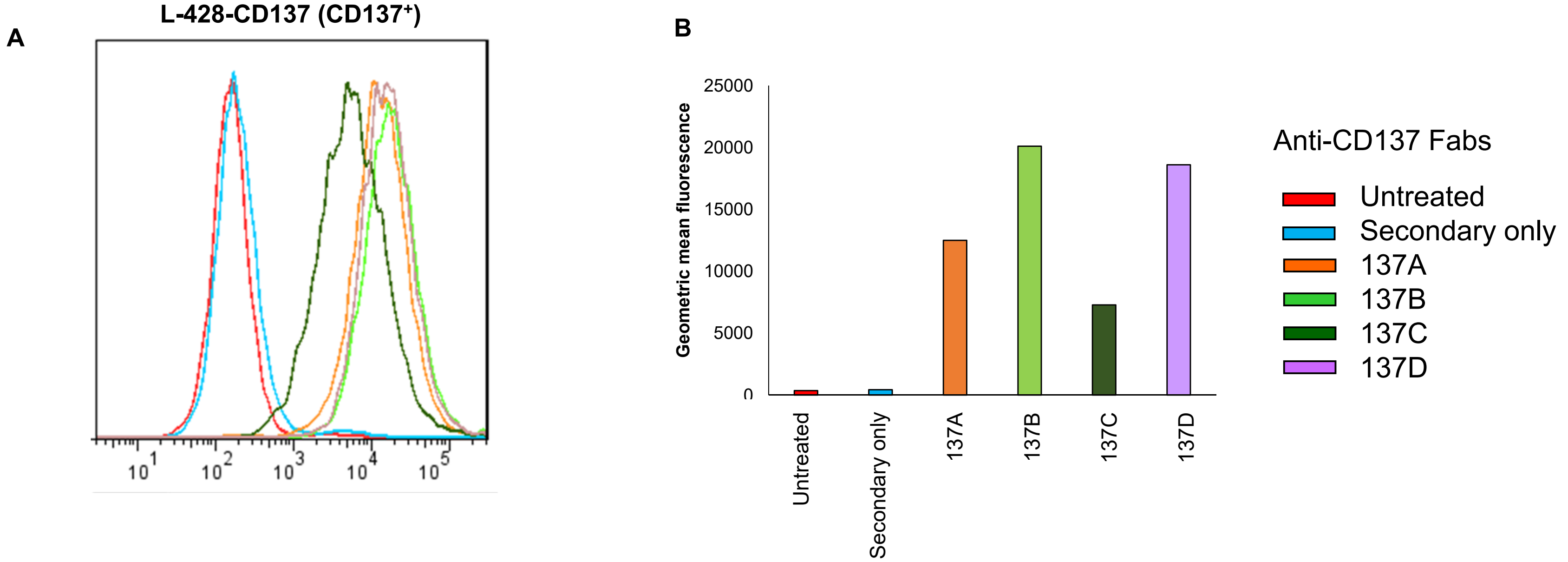

Binding of anti-CD137 Fabs to CD137[+] HL cell line (L-428-CD137).
5×10^5 L-428-CD137 cells were treated with 10 µg/ml of anti-CD137 Fabs and detected using anti-Fab-AF647. (A) Histogram of anti-CD137 Fab binding to L-428-CD137 cells. (B) Geometric mean fluorescence of anti-CD137 Fab binding. Data representative of 2 independent experiments.

Supplementary Figure 4

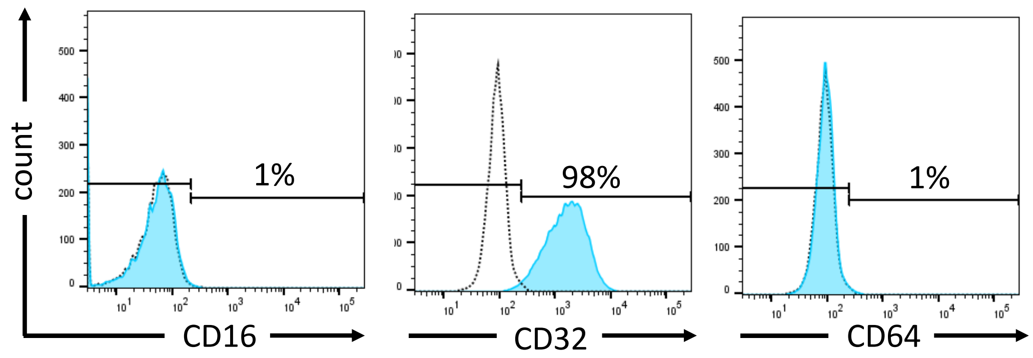

Expression of Fcgamma receptors on KM-H2 cells.
Expression of Fcgamma receptors on KM-H2 cells was determined by flow cytometry. Open histogram, unstained; blue histogram: CD16, CD32 or CD64. The numbers in panels indicate the percentages of positive cells.
